# The difference in heat transport characteristics of the heart and lung meridians: A comparative study of COPD patients and healthy subjects

**DOI:** 10.1097/MD.0000000000023804

**Published:** 2021-02-05

**Authors:** Xiaoyu Li, Yongliang Jiang, Hantong Hu, Yajun Zhang, Jiali Lou, Xiaofen He, Jing Sun, Yuanyuan Wu, Junfan Fang, Xiaomei Shao, Jianqiao Fang

**Affiliations:** a The Third Affiliated Hospital of Zhejiang Chinese Medical University, Hangzhou City, Zhejiang Province; b Department of Neurobiology and Acupuncture Research, The Third Clinical Medical College, Zhejiang Chinese Medical University, Key Laboratory of Acupuncture and Neurology of Zhejiang Province, Hangzhou, China.

**Keywords:** chronic obstructive pulmonary disease, infrared thermography, meridians, site specificity

## Abstract

**Background:**

The vast majority of previous studies focused on the relationship between 1 meridian and 1 organ, and the comparison and specificity between 2 meridians is rarely explored. Thus, the aim of this study is to compare the heat transport characteristics between 2 different meridians and the specificity between them will also be investigated.

**Methods:**

The Lung and Heart meridians are chosen for comparison of 2 different meridians. We will enroll 120 subjects and divide them into the healthy control group, chronic obstructive pulmonary disease (COPD) group and healthy intervention group, in a 1:1:1 ratio. Infrared thermography (IRT) will be used to assess the heat transport characteristics of the Heart and Lung meridians. The specificity for the meridian-visceral association will be investigated by comparing the difference in heat transport characteristic between the Heart and Lung meridians in the healthy control group and COPD group. Meanwhile, moxibustion will be given to subjects in the Heart meridian and Lung meridian respectively in the healthy intervention group to verify the specificity for the surface-surface association.

**Results:**

The primary outcomes will be the temperature of corresponding sites along the Heart and Lung meridians.

**Conclusion:**

This study will verify the specificity between different meridians by comparing the difference in heat transport characteristic. The findings will guide the selection of acupoints to optimize the therapeutic effect of acupuncture and help determine whether IRT could be used to assist in the diagnosis of COPD.

**Ethics and dissemination:**

The study has been approved by the Third Affiliated Hospital of Zhejiang Chinese Medical University (Approval No. ZSLL-KY-2019-001G-01).

**Trial registration numbers:**

NCT04046588.

## 1 Introduction

Acupuncture is a non-pharmacological therapy in traditional Chinese medicine, which involves the insertion of needles into specific points into the body surface to treat a wide range of diseases. It has attracted increasing worldwide interest in recent years. Meridian theory is the guidance of acupuncture practice and it regards that meridians distribute across the human body, thereby connecting the body surface with internal organs. Although the essence of meridian remains unknown, the specific biological characteristic of meridians is generally acknowledged and has been intensively investigated in past few years. Various modern techniques have been applied to investigate relevant biological characteristic of meridians, such as bio-impedance spectroscopy,^[1–3]^ near-infrared spectroscopy,^[4–6]^ laser Doppler flowmetry,^[7–9]^ and infrared thermography (IRT).^[10,11]^ However, the vast majority of previous studies focused on the relationship between 1 meridian and 1 organ, and the specificity between 2 meridians is rarely explored.

Based on the meridian-visceral association in meridian theory, the change of biological characteristic (including temperature) in particular sites of the relevant meridian could reflect the pathological state of the affected internal organ. Similarly, modern sciences have found that abnormal body temperature in specific sites is a indicator of illness.^[12]^ IRT is a modern technique to monitor body temperature, so it has been widely used in the discovery and diagnosis of a wide range of diseases, such as tumors, breast diseases, orthopedic diseases, endocrine diseases, and peripheral vascular disorders.^[13–15]^ It also has advantage of non-invasiveness, convenience, continuity and reproducibility to map the temperature distribution of the body surface remotely.^[16]^ Therefore, IRT was adopted to assess and compare the heat transport characteristics between 2 different meridians in this study.

The aim of the present study is to evaluate and compare the heat transport characteristics between 2 different meridians. Meanwhile, we will investigate the specificity for these 2 meridians. Specifically, the Lung and Heart meridians are selected for comparison. Thus, chronic obstructive pulmonary disease (COPD) patients and healthy adults will be enrolled as research subjects. The results of this study will provide new scientific explanations to the empirical facts and laws that still have significant clinical value in the theory of meridians and will be of great significance to the reconstruction of the current meridian theory and the development of acupuncture.

## 2 Methods

### 2.1 Study design

**Figure 1:**
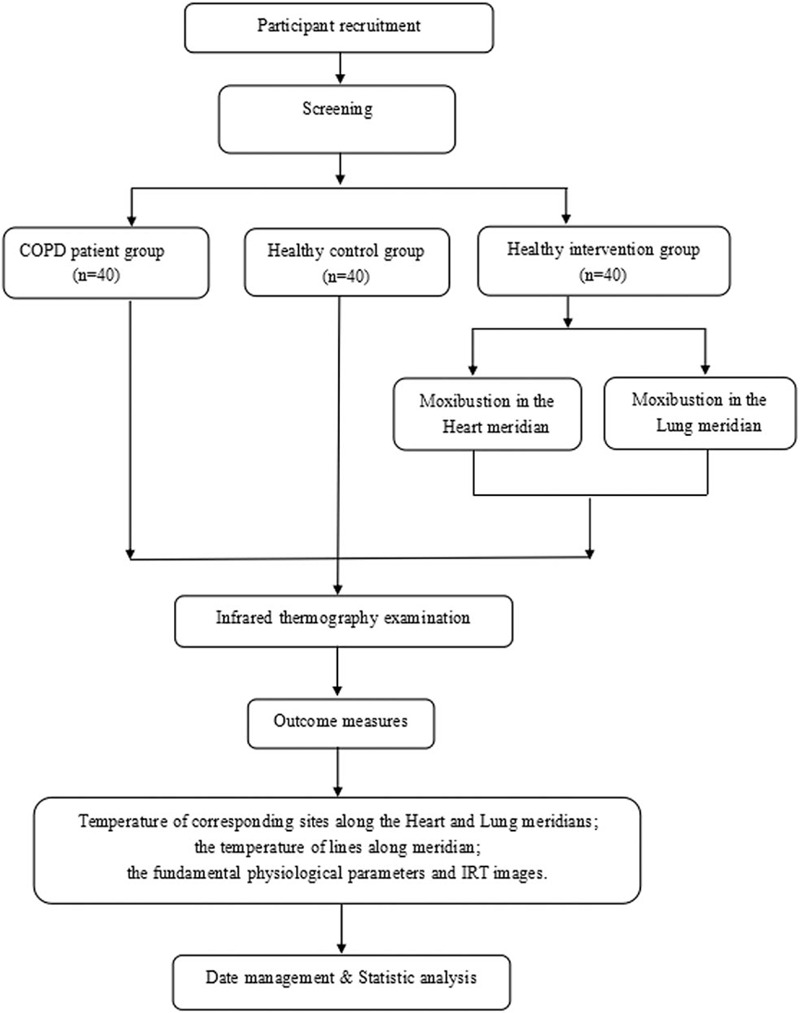
Study design flowchart. Note: COPD = chronic obstructive pulmonary disease.

This is a single-center, open-label, prospective and case-controlled clinical trial with an allocation ratio of 1:1 conducted in the Third Affiliated Hospital of Zhejiang Chinese Medical University. All participants will be divided into the healthy control group, COPD group and healthy intervention group. A flow diagram of the trial is shown in Figure 1 and Table 1.

**Table 1:** Schedule for enrolment, IRT examination and assessments.

		Study stage	
Time points		Screening stage	IRT examination stage
Enrolment	Eligibility screen	○	
	Informed consent	○	
	Allocation	○	
	Collect demographic data	○	
	Check vital signs	○	○
	Check medical report	○	
IRT examination and assessments	IRT examination		○
	Moxibustion (only for the healthy intervention group)		○
	Adverse event assessment		○
	Compliance assessment		○
	Recording of combined medications during the study		○

### 2.2 Sample size estimation

This trial is a meridian study using IRT to assess the heat transport characteristics of 2 different meridians. No existing references could be used to calculate the sample size. Thus, the sample size estimation is mainly based on feasibility and the funds of our trial. Finally, 120 participants will be included, with 40 subjects in each group respectively.

### 2.3 Participants

The subjects of this study will include healthy volunteers and COPD patients.

#### 2.3.1 Diagnostic criteria for COPD

The diagnostic criteria of COPD is based on “the diagnosis and treatment guidelines of COPD” by the Thoracic Society of Chinese Medical Association in 2013 and “the Global Initiative for Chronic Obstructive Lung Disease (GOLD)” in 2017.^[17,18]^ Clinical symptoms include dyspnea, chronic cough and expectoration. Patients often have a history of exposure to various risk factors. And persistent airflow obstruction is indicated by the pulmonary function test (post-bronchodilator FEV1 /FVC<0.70). In addition, other possible diseases are excluded.

#### 2.3.2 Inclusion criteria

##### 2.3.2.1 Inclusion criteria for healthy volunteers

1. Healthy volunteers should provide a recent medical examination report to confirm they have not any cardiovascular, respiratory, digestive, urinary, hematological, endocrine and neurological disease;
2. 35≤ age ≤75 years, male or female;
3. Participants have clear consciousness and could communicate with others normally;
4. Participants could understand the full study protocol and written informed consent is provided by themselves or their lineal kin.

##### 2.3.2.2 Inclusion criteria for the COPD patients

1. Patients should meet the above diagnostic criteria, and the severity of COPD is in the stage of GOLD 2 or 3 based on pulmonary function testing;
2. COPD patients in the stable phase, who present with mild symptoms of cough, expectoration and short breath;
3. 35≤age ≤75 years, male or female;
4. Patients have clear consciousness and could communicate with others normally;
5. Patients could understand the full study protocol and written informed consent is signed.

#### 2.3.3 Exclusion criteria

##### 2.3.3.1 Exclusion criteria of the healthy volunteers

1. Participants have mental illness, severe depression, alcohol dependence or history of drug abuse;
2. Pregnant or lactating participants;
3. Participants are participating in other trials.

##### 2.3.3.2 Exclusion criteria for the COPD patients

1. Patients who fail to meet the diagnostic criteria for COPD, or COPD patients in the phase of acute exacerbation;
2. Patients have the following complications, such as bronchial asthma, bronchiectasis, active tuberculosis, pneumothorax, chest trauma, tumors of the lung or thorax;
3. Patients have concomitant Heart diseases;
4. Patients have serious concomitant diseases of major system and fail to treat them effectively, such as diseases of the digestive, urinary, respiratory, hematological, and nervous system;
5. Patients have mental illness, severe depression, alcohol dependence or history of drug abuse;
6. Pregnant or lactating patients;
7. Patients are participating in other trials.

### 2.4 Recruitment procedures

All the participants will be enrolled from the Third Affiliated Hospital of Zhejiang Chinese Medical University. Our study will be advertised on the Internet and on posters in communities and hospitals. All participants will be informed of the purpose, contents, benefits and potential harms of the study before signing the written informed consent.

### 2.5 Blinding

The participants, acupuncturists and outcome assessors will not be blinded. In the data analysis stage, blinded statistical analysis will be adopted.

### 2.6 Intervention

All the participants are requested to refrain from consuming tea, alcohol, coffee and smoking on the examination day. Besides, food and exercise are also forbidden within 1 hour before the IRT examination.

#### 2.6.1 Examination environment

An experimental room is set up and the temperature for the examination environment is within 23 to 25°C. The relative humidity is controlled between 30% and 40%. There is no direct sunlight and obvious air convection in the experimental room.

#### 2.6.2 Procedures for the IRT examination and moxibustion intervention

A thermograph (NEC InfRec R450, Avio Infrared Technologies Co., Ltd., Tokyo) will be used to record thermal images for evaluating the heat transport characteristics of the Heart and Lung meridians. The participants will be requested to stabilize for 15 minutes in a supine position to adapt to the room temperature prior to IRT examination. They are also informed to keep silent and normal breath and avoid limb movement during the full measuring period.

The height and angle of the infrared thermal imaging camera will be adjusted so that the measurement sites of the participant's left arm is located in the center of the camera screen. When the size is moderate in the screen, thermal images will be recorded. All thermal images will be analyzed by the software InfRec Analyzer NS9500 (Avio Infrared Technologies Co., Ltd., Tokyo), which could display different temperature in various colors and select points or sites for temperature measurement. The temperature of corresponding acupoints or sites on the Heart and Lung meridians will be analyzed.

##### 2.6.2.1 Healthy control group and COPD group

For participants in the COPD group and healthy control group, the IRT examination of the measurement sites will last for 5 minutes, with 1 thermal image taken every 1 minute. For each thermal image, we measure the temperature of corresponding acupoints or sites and then averaged them. The baseline temperature of the Heart and Lung meridians will be measured.

##### 2.6.2.2 Healthy intervention group

For participants in the healthy intervention group, 2 sessions of moxibustion intervention will be performed in the Heart meridian and Lung meridian successively. The IRT examination of the measurement sites will last for 25 minutes, including 5-minutes baseline, 15-minutes moxibustion intervention and 5-minutes post-stimulation recording, with 1 thermal image taken every 1 minute.

Intervention in the Heart meridian: By igniting the moxa stick and inserting it into a homemade moxibustion holder to adjust appropriate angle and height, moxibustion will be performed above acupoint Shaohai (HT3) of the Heart meridian for 15 minutes. During moxibustion, the temperature of the Heart meridian and the Lung meridian will be measured.

Intervention in the Lung meridian: The moxibustion acupoint is Chize (LU5) of the Lung meridian. The IRT examination, moxibustion procedure and measuring time points are the same as 1).

#### 2.6.3 Locations of the acupoints

HT 3: On the anteromedial aspect of the elbow, just anterior to the medial epicondyle of the humerus, at the same level as the cubital crease.
LU 5: On the anterior aspect of the elbow, at the cubital crease, in the depression lateral to the biceps brachii tendon.

#### 2.6.4 Concomitant treatments

Throughout the study phase, all subjects in the COPD group will maintain their previous treatments. If add-on treatments are adopted during the study course, the details should be documented.

Meanwhile, participants in the healthy control group and healthy intervention group should not take any medications during the study. If medications or other therapies are adopted given to sudden diseases, relevant information should be documented.

### 2.7 Outcome measures

Primary outcomes will be the temperature of corresponding points along the Heart and Lung meridians and the temperature of lines along meridian, which represents the average temperature of the Heart meridian and Lung meridians in the forearm. Secondary outcomes will be included the fundamental physiological parameters (systolic blood pressure, diastolic blood pressure, respiratory rate, and body temperature) and IRT images.

### 2.8 Safety assessment

Adverse events related to IRT examination or moxibustion will be recorded and evaluated by the researchers throughout the trial. If serious adverse events happen, the researchers should inform the ethics committee immediately, who will decide on whether the participant should be withdrawn from the study.

### 2.9 Quality control

This protocol has been modified based on suggestions of experienced acupuncturists. All researchers will undergo training on the operating procedures of the trial. Monitors will verify case report forms and the IRT examination during the study. Dropouts and withdrawals will be documented in details throughout the trial. Economic compensation will be adopted to improve compliance and reduce dropouts. Participants’ information will be stored in locked file cabinets at the study sites with limited access; only investigators have the right to access the data.

### 2.10 Data collection and statistical analysis

SPSS 17.0 (SPSS Inc., Chicago, IL, USA) will be used to perform statistical analysis by third party statisticians blinded to this trial. Numeric data with normal distribution will be expressed as mean ± standard deviations, whereas data with skewed distribution as median and inter quartile ranges.

Between-group differences for baseline dichotomous variables will be tested using the Chi-Squared test. We will use repeated measures ANOVA to assess change in continuous variables before and after intervention and paired samples *t*-test to compare the changes within the groups, whereas the independent samples *t*-test will be employed for comparisons between the groups. Within-group or between-group comparison for data with skewed distribution is assessed using non-parametric test. Statistical significance is defined as the *P* value <.05.

### 2.11 Patient and public involvement

Patients and public are not involved in the design of this study.

### 2.12 Ethics approval

Ethics approval (number: ZSLL-KY-2019-001G-01) was obtained from the Third Affiliated Hospital of Zhejiang Chinese Medical University. Prior to the trial, all subjects will be fully informed about contents concerning the objective, benefits and potential risks of the trial. They have full rights to decide whether to participate in the trial. Informed consent should be signed if they participate in this trial.

The trial was registered at Clinical Trial Registry with the identification code NCT04046588. The study findings will be disseminated through presentation at peer-reviewed journals, with online access. We also to plan to present them in select conferences and scientific meetings.

## 3 Discussion

Previous studies have proved the specific biological characteristic of meridians. Among them, the heat transport characteristics is one of the hotspots of meridian research. Phenomenon of high temperature lines along the meridians and its related mechanisms are the focus of meridian studies involving IRT.^[19]^ Some scholars used IRT to observe the temperature changes before and after acupuncture at acupoints (Hegu) and non-acupoints (epidermis and muscles).^[11]^ As a result, the temperature of the midpoint of the nail bed and the midpoint of the metacarpal bones of each finger were found significantly increased after acupuncture, and this phenomenon continued until the end of 30-minutes acupuncture, while the temperature at each analysis point in the control group did not increase, thereby confirming that acupuncture could induce the local high temperature lines of the meridian. However, whether the induced local high temperature line is consistent with the classical meridian circulation line in meridian theory requires further verification.

Bedsides, according to the theory of acupuncture, there is a close relationship between the external reaction of the body surface and changes of internal organs. When specific internal organ is kept in a pathological state, changes in biological characteristics, such as electrical,^[20,21]^ thermal,^[22,23]^ and optical features,^[24,25]^ could be presented on the body surface of related meridians. Among them, the change in heat transport characteristics of corresponding meridian is one of the pathological manifestations of visceral diseases. Overall, previous meridian studies involving IRT have revealed 2 important findings. On one hand, the acupoints and meridians tend to have relatively higher temperature. It reflects more special physiological functions and carries important physiological and pathological information.^[26,27]^ On the other hand, during the switch from the physiological state to pathological state, corresponding acupoints of meridians will manifest relevant changes in heat transport characteristics.^[28,29]^

Nevertheless, the vast majority of previous meridian studies focused on 1 meridian and 1 organ, the correlation between multiple meridians and multiple organs is rarely explored. Therefore, our study selects the Heart and Lung meridians for comparison. Consequently, COPD patients and healthy adults are chosen as research subjects. By using IRT to assess and compare the heat transport characteristics between the Heart and Lung meridians, the specificity between 2 different meridians in physiological and pathological states will be investigated, respectively. The present study has several strengths as below.

Firstly, this trial is the first meridian study to investigate and compare the heat transport characteristics between 2 different meridians by using IRT, which is a mature technique to measure the heat transport characteristics in acupuncture-related trials,^[30–32]^ owing to its strengths of the accessibility, clarity and objectivity of temperature measurements. It uses the human body as a source of thermal radiation, uses special sensing probes to detect near-infrared radiation from the human body surface, and then passes a specific signal processing program to convert the invisible near-infrared spectrum into visible infrared thermal spectrum. The heat balance of human skin temperature can be maintained by activities of skin, internal tissue, local blood circulation, metabolic activity, circadian rhythm, sympathetic and parasympathetic nervous system, which is the physiological basis of IRT for medical monitoring.^[33]^ Compared with most techniques used in meridian studies, IRT has particular advantage of a linear analysis of the entire meridian routes instead of just acupoint analysis. Given to that previous meridian research mainly used IRT for TCM syndrome research or acupoint specificity study,^[34,35]^ it is refreshing that the purpose of this clinical trial is to investigate the heat transport characteristics of meridian phenomena by using an objective assessment tool and verify the site specificity between 2 specific meridians in patients with COPD and healthy adults. For 1 thing, we assumed that there is a more significant change in the heat transport characteristics of the Lung meridian between the healthy control group and COPD group. By comparing the heat transport difference between the Heart and Lung meridians in the healthy control group and COPD group, the site specificity for the meridian-visceral association will be verify. For another thing, we assumed that there is a more significant change in heat transport characteristics in relevant sites of the stimulated meridian between the stimulated meridian and the non-stimulated meridian in the healthy control group. By performing moxibustion in the Heart meridian and Lung meridian respectively in the healthy control group and COPD group, surface-surface association between 2 specific meridians, the heat transport change will be compared between the Heart and Lung meridians and the site specificity for the surface-surface association will be explored. In brief, with a comparison of the healthy and COPD groups, we sought to confirm whether the meridians were sensitized in a pathological state. As well, by performing the moxibustion intervention on the healthy group rather than the COPD group, we sought to confirm whether the meridians were also activated in a physiological state. This is an important question that needs to be addressed in meridian research.

Secondly, this study will provide a basis for IRT to assist clinical diagnosis of COPD and further treatment. In this study, we will collect the heart transport data of the Heart and Lung meridians in physiological and pathological states to observe whether the distribution characteristics of infrared images of COPD patients are significantly different from healthy subjects. Meanwhile, we will explore whether the temperature of the relevant acupoints in patients with COPD on the lung and Heart meridians show significant abnormalities. According to the meridian theory, the illness of organs could result in the appearance of sensitized points on the body surface, which is also an optimal target for acupuncture treatment.^[36,37]^ Thus, findings of this study will guide the clinical selection of acupoints to obtain the best therapeutic effect of acupuncture by exploring the acupoints most relevant to COPD.

Nevertheless, we must address several limitations of the present study. Firstly, because the skin temperature of acupoints could be affected by a variety of external factors.^[38]^ Although this study has minimized the influence of external factors on temperature (e.g., controlled environment temperature or humility, adequate rest prior IRT examination), the difference in temperature caused by individual differences cannot be completely avoided. Secondly, due to limited time and funds, it is not possible to conduct a large sample study. Therefore, this study uses a small sample size, and we look forward to expanding the sample size in the future.

## Author contributions

**Conceptualization:** Jianqiao Fang.

**Data curation:** Yajun Zhang, Jiali Lou.

**Investigation:** Xiaoyu Li, Hantong Hu.

**Methodology:** Yongliang Jiang, Hantong Hu, Junfan Fang.

**Project administration:** Jianqiao Fang.

**Software:** Xiaoyu Li, Hantong Hu, Jing Sun.

**Supervision:** Yuanyuan Wu.

**Validation:** Yuanyuan Wu, Xiaomei Shao.

**Visualization:** Xiaofen He.

**Writing – original draft:** Xiaoyu Li.

**Writing – review & editing:** Yongliang Jiang, Hantong Hu.
